# A neural computational model for bottom-up attention with invariant and overcomplete representation

**DOI:** 10.1186/1471-2202-13-145

**Published:** 2012-11-29

**Authors:** Zou Qi, Zhao Songnian, Wang Zhe, Huang Yaping

**Affiliations:** 1 Department of Computer Science, Beijing Jiaotong University, Beijing, 100044, China; 2 LAPC, Institute of Atmospheric Physics, Chinese Academy of Sciences, 100029, China; 3 Institute of High Energy Physics, Chinese Academy of Sciences, Beijing, 100049, China

## Abstract

**Background:**

An important problem in selective attention is determining the ways the primary visual cortex contributes to the encoding of bottom-up saliency and the types of neural computation that are effective to model this process. To address this problem, we constructed a two-layered network that satisfies the neurobiological constraints of the primary visual cortex to detect salient objects. We carried out experiments on both synthetic images and natural images to explore the influences of different factors, such as network structure, the size of each layer, the type of suppression and the combination strategy, on saliency detection performance.

**Results:**

The experimental results statistically demonstrated that the type and scale of filters contribute greatly to the encoding of bottom-up saliency. These two factors correspond to the mechanisms of invariant encoding and overcomplete representation in the primary visual cortex.

**Conclusions:**

(1) Instead of constructing Gabor functions or Gaussian pyramids filters for feature extraction as traditional attention models do, we learn overcomplete basis sets from natural images to extract features for saliency detection. Experiments show that given the proper layer size and a robust combination strategy, the learned overcomplete basis set outperforms a complete set and Gabor pyramids in visual saliency detection. This finding can potentially be applied in task-dependent and supervised object detection.

(2) A hierarchical coding model that can represent invariant features, is designed for the pre-attentive stage of bottom-up attention. This coding model improves robustness to noises and distractions and improves the ability of detecting salient structures, such as collinear and co-circular structures, and several composite stimuli. This result indicates that invariant representation contributes to saliency detection (popping out) in bottom-up attention.

The aforementioned perspectives will significantly contribute to the in-depth understanding of the information processing mechanism in the primary visual system.

## Background

Bottom-up attention has been widely studied in physiology, psychology, neural science and computer vision. It is usually attributed to early vision, such as to the manner by which the primary visual cortex (V1) encodes low-level features and forms a saliency map. Numerous studies have explored theories and computational models to provide an efficient input to the saliency detection. For example, Treisman and Gelade [1] developed the feature integration theory to explain how primary visual features are processed and represented with separate feature maps and are later integrated into saliency maps. Koch and Ullman [2] proposed a biologically plausible computational framework to model the process that attracts focus-of-attention to the most conspicuous areas. Several other hypotheses were tested, including a saliency map created in the primary visual cortex that does not need any feature combination by Zhaoping Li [3], and those models motivated by an imitation of information processing mechanisms in the V1 [4,5]. In addition, the effects of overcomplete bases on encoding a bottom-up saliency map is of current interest [6,7]. While more and more neurobiological properties of the V1 have been accepted, the way they modulate saliency detection remains unclear [8]. Models limited to simple cell simulations cannot sufficiently satisfy neurobiological constraints [9]. The receptive fields of simple cells are too small to place competing stimuli inside them, which is an important condition for attentional experiments [10]. Thus, attentional modulation is most prominent in higher cortical areas, where receptive fields are wide and where several objects can compete inside a single receptive field. Simple cells alone contribute less strongly to selective attention, especially for complex tasks (conjunctive search). The involvement of simple cells with other mechanisms in the primary visual cortex, such as complex cells or synchronised oscillation, to account for the pre-attentive process seems to be more biologically plausible. The effects of other mechanisms beyond simple cells on bottom-up attention are worth exploring.

Recently, deep learning by a hierarchical network has been thoroughly researched and successfully used in object recognition [11,12]. The approach is regarded as an ideal model to simulate the information representation of humans. It provides a hierarchical learning mechanism that gains invariant representation, and can be generalised to include other coding strategies.

**Figure 1:**
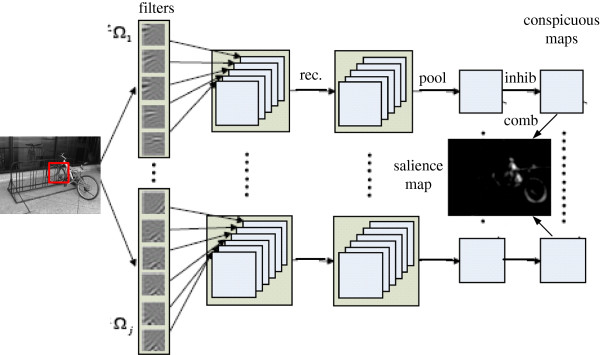
**Diagram of our model.** An image is first convolved with each group of filters in the first layer, where the filters are organized into a topographic array (filters and topography are learned by the PCICA algorithm beforehand). Outputs of that are rectified by the sigmoid and the absolute functions. Then, in the second layer, the rectified outputs within each group are pooled to produce invariant representation, which is subjected to the inhibition simulated by the convolution with the DoG function. Conspicuous maps from different groups are combined to produce a final saliency map as output.

This paper aims to introduce deep network into saliency detection and to investigate the influences of different coding factors. We discuss two central questions and a series of related ones. First, does invariant representation in the V1 affect bottom-up attention? If so, what is the optimal network structure to obtain invariance? Second, what is the effect of overcomplete representation on saliency detection? Another issue is the concomitant feature combination on such a massive scale. The overall scheme of our model is given in Figure 1.

### Traditional bottom-up attention models vs. our model

Bottom-up attention models extract multi-dimensional features from an image and combine these features into a saliency map where the most salient object will be perceived.

In the feature extraction stage, computational models motivated by imitation of the primary visual cortex often use Gabor filters to extract orientation information at different scales. Properties of Gabor filters resemble simple cells’ receptive fields and can provide input to the bottom-up saliency map. Similar methods also use Gaussian pyramids [13], Fourier transformation, or wavelets decomposition [14] to extract features similar to the responses of cells. One of representative models proposed by Itti et al. [4] adopted Gaussian pyramids to extract color, intensity, and orientation features at different levels. Grigorescu et al. [15] simulated complex cells and nonclassical receptive field inhibition to detect salient contours. By pooling the responses of two simple cells with orthogonal phases, complex cells are insensitive to exact positions of edges. Nonclassical receptive field inhibition benefits by suppressing homogeneous texture and thus salient contours are perceived easily.

Our model differs from these traditional attention models in several aspects. First, we learn overcomplete basis sets from natural images to extract features for saliency detection, while they directly use Gabor function or other wavelets as filters. Second, we design a hierarchical architecture to learn some invariance, while most attention models use a single layer to perform filtering and thus do not consider any invariance except the invariance to positions. The invariance to positions is obtained in some models [15] by summing energies of a pair of Gabor functions with orthogonal phases. Our model learns a broader range of invariance by first obtaining the overcomplete topological basis set and then pooling the responses of topological bases in the neighborhood. Third, our model can detect global salient structures besides local salient points. These two different kinds of saliency detection are related with the definitions of saliency in space-based attention and object-based attention.

Computationally, an explicit representation of saliency in most models implements centre-surround differences. A type of local spatial selection is presumed to be necessary for preattentive feature detection [4]. Neurons in the retina, lateral geniculate nucleus, and in the early visual cortical areas are tuned to local contrast such as intensity contrast and colour opponency [9]. For example, the response of a retinal neuron tuned to the intensity of the centre-surround contrast can be computed by convolving the luminance channel of the input image by a Difference of Gaussians (DoG) filter. Another view of saliency is provided with a global structure rather than with local points. Typically, Gestalt psychologists defined saliency as whether a structure respects certain perceptual organisation rules such as proximity, good continuity, and closure [16]. Once local primitives form a structure satisfying these rules, they will be perceived as a whole. In the experiments, we tested our model using two kinds of saliency detection tasks.

### V1 coding models

Coding models simulating information processing mechanism in the V1 are often motivated by an imitation of the properties of simple cells and complex cells. The Gabor function is regarded as best describing the classical models of simple cells and complex cells. A single Gabor wavelet is similar to the receptive field of a simple cell. The square sum of the responses of a pair of Gabor wavelets with orthogonal phases is similar to that of a complex cell.

However, this classical picture is incomplete. Cells in V1 show much more receptive field properties compared with what this simple model can explain. For example, they show end-inhibition [17,18] and over-representation of the orientation and frequency [19].

Several coding models learned statistical properties from natural images and developed receptive field properties that match those of cortical cells to describe the complete picture. Hyvarinen and Hoyer proposed the independent subspace analysis (ISA) [20] and the topographic independent component analysis (TICA) [21]. Both models can extract the phase invariant and shift invariant features similar to the responses of complex cells. Most recently, deep learning [11,12] was deeply researched and successfully used in object recognition. Wang et al. [22] developed a more computationally efficient model based on pairwise cumulant based methods for independent component analysis (PCICA). It captured the topological relationships by the pairwise cumulant to obtain results similar to ISA and TICA with fast convergence. Obtaining invariance by pooling is not new for object recognition. Some works discussed advantages and disadvantages of different pooling operations [12], but this is beyond our topic here.

Overcomplete representation is another important property in the primary visual cortex. Despite our recognition of its usefulness in early vision, we have not fully understood its role in forming a saliency map [7]. Information processing in V1 provides input to all subsequent cortical areas, to fully represent a complex scene and satisfy different needs. To describe all features including structure and details at different levels, the amount of basis vectors should be large. Several coding models considering overcomplete basis sets have been proposed, such as TICA and OPCICA (Overcomplete Pairwise Cumulant based methods for Independent Component Analysis). OPCICA is successfully used in object recognition. Recently, some saliency detection models using matrix decomposition learned overcomplete bases from color images [6]. Although it uses sparse coding and overcomplete bases, it is less biologically motivated and more mathematically implemented.

## Results and discussion

To investigate the influences of different coding factors on bottom-up attention, we compared the results of two kinds of datasets: synthetic images widely used in cognitive psychological experiments on visual attention and natural images. The experimental environment was simulated in MATLAB that ran under Intel Core i5 2.66 GHz CPU. The details of the training and testing processes are summarized as follows:

### Training

1. obtaine 50,000 image patches of 16×16 from the training dataset and preprocess them with whitening and dimension reduction.

2. initialize the basis set to be a random matrix and orthogonalize it.

3. update the basis set according to the rule defined in the PCICA algorithm.

In training, we used the grey image dataset^a^, which is the standard dataset used in ICA and in sparse coding models to learn an overcomplete basis set by the PCICA.

### Testing

1. Using the learnt overcomplete topological bases by the PCICA, we extracted primitive features by convolving an input image with each filter.

2. The outputs in step (1) were then rectified by the sigmoid and the absolute function.

3. The primitive features, when in step (2), were pooled and refined to form invariant features descriptors. As the PCICA algorithm has organized the bases into a topographic array, pooling is defined in a neighbourhood by subsampling. That is, horizontal and vertical intervals between two neighbourhoods/pools are constants. The pooling in this study was implemented by the square root of the sum of the squares of those units belonging to the same pool. Refinement was implemented by computing the similarity (according to formula 6) between pairs of filter responses and then selecting the ones whose similarity exceeded the threshold.For the model without invariant feature coding, this pooling was not needed because the conspicuous maps were obtained by directly performing DoG suppression on the feature maps from step (2).

4. For the model with invariant feature coding, the conspicuous map was obtained by performing surround inhibition on each invariant feature map when in step (3). The surround inhibition was implemented as a convolution of the invariant feature with the DoG (formula 9).

5. The conspicuous maps were combined into a final saliency map by iteration strategy (formula 11). When the number of conspicuous maps was large, we used the K-means algorithm to organise the maps into N clusters, iterated each cluster centre according to formula (11), and then obtained the saliency map by summing up the N iterated results.

**Figure 2:**
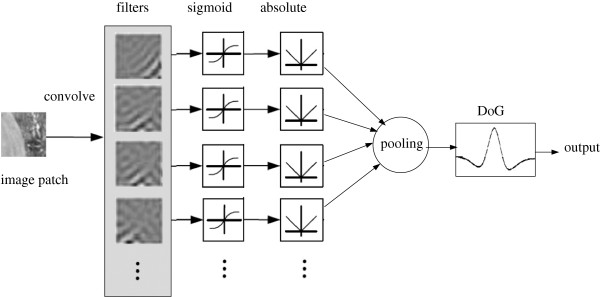
**Diagram of the fully connected network.** An image is first convolved with all filters in the first layer, the outputs of which are rectified by the sigmoid and the absolute functions. Then, all the rectified outputs are pooled in the second layer to produce a single feature map, which is subjected to the inhibition simulated by convolution with the DoG function. The final output is the saliency map.

**Figure 3:**
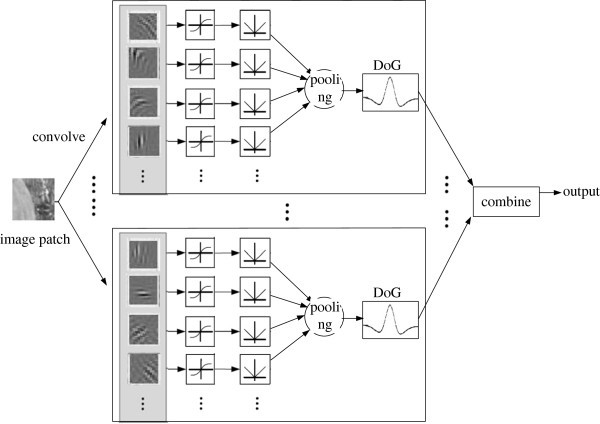
**Diagram of the randomly connected network.** An image is first convolved with each random group of filters in the first layer (the number of filters in a group is fixed but which filters belong to a group are random), the outputs of which are rectified by the sigmoid and the absolute functions. Then, in the second layer, the rectified outputs within each random group are pooled to produce a single feature map, which is subjected to the inhibition simulated by the convolution with the DoG function. Feature maps from different random groups are combined to produce a final saliency map as output.

We also build a fully connected network (Figure 2) and a randomly connected network (Figure 3) for a more detailed comparison.

### The optimal network structure for invariant representation

For experiments in this part, we learned 196 filters of size 16×16 by PCICA. The filter size was changed to 8×8, 12×12 and 20×20 when we compared the models performances under varying layer 1 RF sizes. Then we got 25 invariant features by pooling filters in 5×5 neighborhoods over the 14×14 topographic array(composed of 196 filters). Two neighborhoods are overlapped by two filters both horizontally and vertically. The neighborhood size was changed to 3×3 and 7×7 when we compared the models performances under varying layer 2 RF sizes.

To make our results comparable to those of the model by Itti and Koch (Saliency Tool), we only selected an orientation channel for Salience Tool to form saliency maps. Salience Tool used the default parameters setting of 72 Gabor filters at 9 scales, 4 orientations(0°, 45°, 90°, 135°), and 2 phases(0°, 90°). It produces 24 feature maps (6 centre-surround difference maps for each orientation angle).

**Figure 4:**
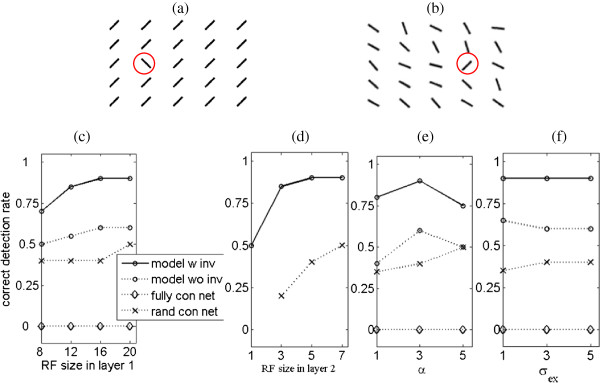
**Saliency detection in visual search where stimuli differ in orientations. (a)** a testing image without orientation noises; **(b)** an example of testing images where orientation noises are in [−30°, 30°] with uniform probability. The target marked by red circles; **(c)** comparison of different models namely the model with invariant features,the model without invariant features, a fully connected network and a randomly connected network, on 20 testing images such as that in (b) when layer 1 RF size varies; **(d)** comparison when layer 2 RF size varies; **(e)** comparison when the coefficient *α*which adjusts the suppression strength varies; **(f)** comparison when excitation bandwidth *σ*_*ex *_varies. See the text for details.

The first experiment is similar to the ”visual search” tasks designed by Treisman and Gelade [1]. According to their results, one target among distractors with orthogonal orientations easily pops out. The tasks become more difficult if orientation noises are added to distracters [7]. We designed distractors with noises between [−30°, 30°] to test the performances of different models under different parameters. The results are shown in Figure 4. 

We generated synthetic images as follows. Target positions and orientations were randomly determined. Distractors orientations were orthogonal to that of the target and were disturbed by orientation noises (in [−30°, 30°] with uniform probability). We generated 20 synthetic images where the target’s position and orientation was random in every image, and showed an example in Figure 4(b). The correct detection rate is defined as the ratio between the times that the maximal in a saliency map refers to the target and the total experimental times.

The fully connected network could not detect the target in all the experimental conditions. The randomly connected network performed better than the fully connected network. The attention model without invariant features coding could produce acceptable results when parameters were tuned properly. The model with invariant features coding performed best though it was also affected by model parameters.

The first layer receptive field (RF) size is highly relevant to the resolution of the image and to texture density. For images with fine and close textures, a small RF size is enough. For images with coarse contours or pieces, the RF size should be larger. As seen in Figure 4, we tested four layer 1 RF sizes, namely 8×8, 12×12, 16×16, and 20×20. As long as the size was greater than the interval between the adjacent bars in Figure 4(b), it would not significantly affect the performance. An RF size of 8×8 was too small, leading to relatively low detection rates. The sizes greater than 16×16 did not produce substantial changes in performance (Figure 4(c)).

We also tested three layer 2 RF sizes, namely 3×3, 5×5, and 7×7. Note that for the model without invariant features, the layer 2 RF size is always 1. For the fully connected network, this size is always the total number of filters in layer 1. Thus, this comparison only works for the model with invariant features and the randomly connected network. For the former, the performance is not affected in such a simple task (Figure 4(d)). However, in the extreme condition, i.e., layer 2 RF size of 1×1 (which is equivalent to the model without invariant features), the correct detection rate drops when orientation noises lie in the [−30°, 30°] range. However, when no orientation noises are added (all distractors are in the same orientation like Figure 4(a)), the correct detection rate of the model without invariant features is the same as that of the model with invariant features. This indicates the layer 2 RF size (the size of pools) influences robustness to noises. For the randomly connected network, correct detection rate rises with the layer 2 RF size.

The coefficient *α*, which adjusts suppression strength, affects performance. Generally, a very small coefficient cannot completely suppress responses to distractors, and a very large one probably suppresses all responses including those to the target. Both of these situations lead to low detection rates (Figure 4(e)). However, a larger *α* results in a better detection rate for the randomly connected network.

Finally, we evaluated the influence of the excitation bandwidth *σ*_*ex *_and the inhibition bandwidth *σ*_*inh *_in the DoG used within the feature competition. For simplicity, we kept the ratio $\frac{\sigma_{\text{inh}}}{\sigma_{\mathrm{ex}}}$ constant, and we only changed *σ*_*ex *_to three values: 1, 3, and 5. This change indicates that *σ*_*ex *_is one, three, and five times as large as the layer 1 RF size. For the model without invariant features, the detection rate dropped with the increase of *σ*_*ex*_. No substantial change was observed for both the model with invariant features and the randomly connected network (Figure 4(f)).

**Figure 5:**
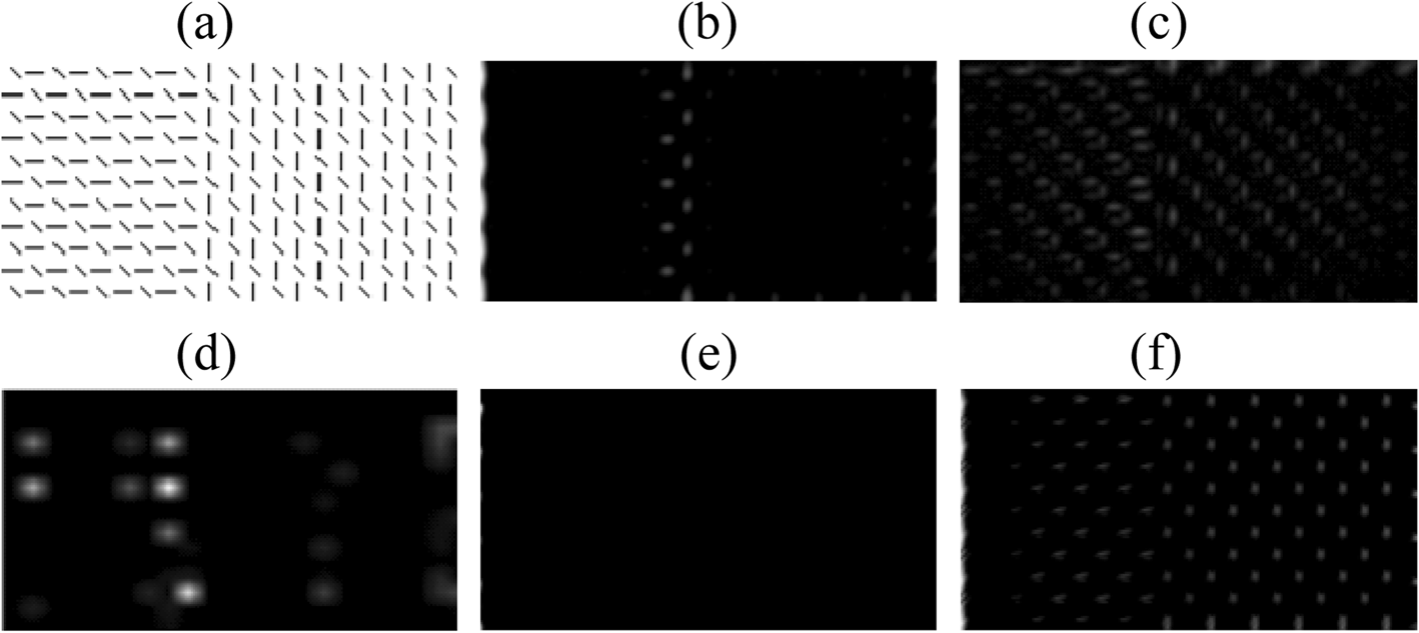
**Saliency detection in composite stimuli. (a)** source image; **(b)** saliency map of the attention model with invariant features coding. When *σ*_*ex *_is 1.8*%*−3*%* of the image size (the larger one between the image width and height),*σ*_*inh *_is three to four times of *σ*_*ex*_, *α*=1.8, saliency detection results are similar to (b); **(c)** saliency map of the attention model without invariant features coding, and parameters do not substantially influence the result; **(d)** saliency map of Itti’s attention model; **(e)** saliency map of a fully connected network; **(f)** saliency map of a randomly connected network. The perceptual interpretation of human observers is reproduced only by our model with invariant features.

The second experiment involved a visual search task in composite stimuli. As shown in Figure 5, the attention model with invariant features outperforms the model without invariant features, fully connected and randomly connected networks, and Itti’s Saliency Tool [4]. The model with invariant features can detect salient objects when parameters vary greatly, whereas the model without invariant features cannot work. The results of our model with several different parameters are shown in Appendix 1. A detailed behavioural analysis for parameter values is also presented. 

**Figure 6:**
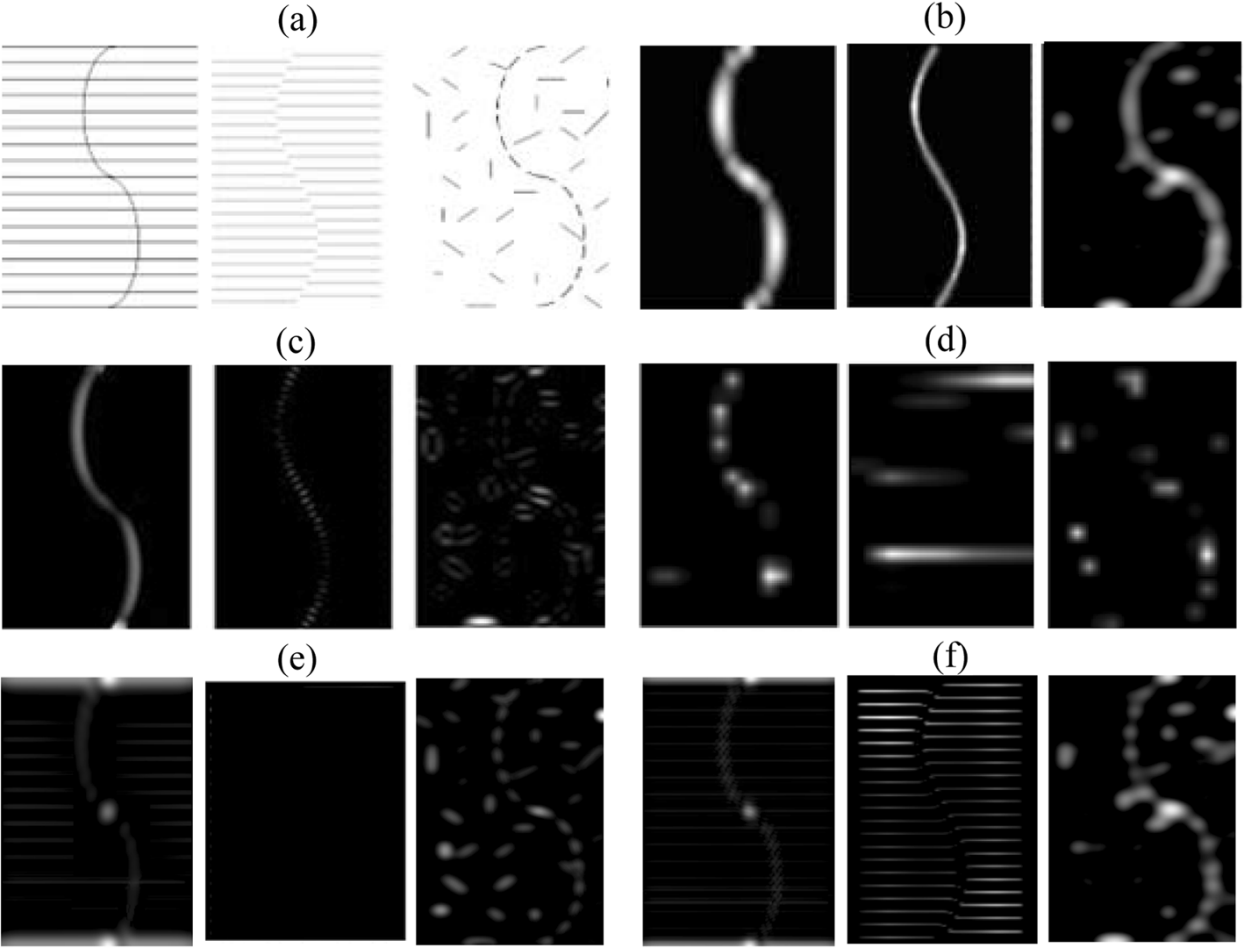
**Detection of global salient structure. (a)** source images; **(b)** saliency maps of the attention model with invariant features coding; **(c)** saliency maps of the attention model without invariant features coding. Results are relatively insensitive to parameters. *σ*_*ex *_is 2% to 5% of the image size (the larger one between the image width and height). *σ*_*inh *_is 4 to 10 times of *σ*_*ex*_, *α*∈[3,4]; **(d)** saliency map of Itti’s attention model; **(e)** saliency map of a fully connected network; **(f)** saliency map of a randomly connected network.

We further tested the models’ performances in detection of global salient structures. As mentioned in the Background section, the global saliency is defined by Gestalt psychologists as whether a structure respects certain perceptual organisation rules such as proximity, good continuity, and closure [16]. The s curve, an illusory contour and a noisy version were taken as testing images. Results are shown in Figure 6. 

As indicated in Figure 6, objects as solid curves are perceived clearly. When objects are illusory contours, the attention model with invariant features coding can detect continuous contours. As parameters vary within the range mentioned in the figure caption, detection results are always continuous contours, while the attention model without invariant features coding detects discrete end points. As parameters vary within the range mentioned in the figure caption, detection results are always discrete ends. When the object is a noisy s curve, differences in saliency detection results between the two attention models are greater. In the model with invariant coding, segments forming the s curve can be detected and most segments forming background can be suppressed. For the model without invariant coding, segments in the background are more salient than those in the s curve. Itti’s Saliency Tool performed better than the attention model without invariant features coding, but for the illusory contour, it performed even worse. We give a detailed comparison and analysis for the fully connected network and the randomly connected network on the illusory contour in Appendix 2.

**Figure 7:**
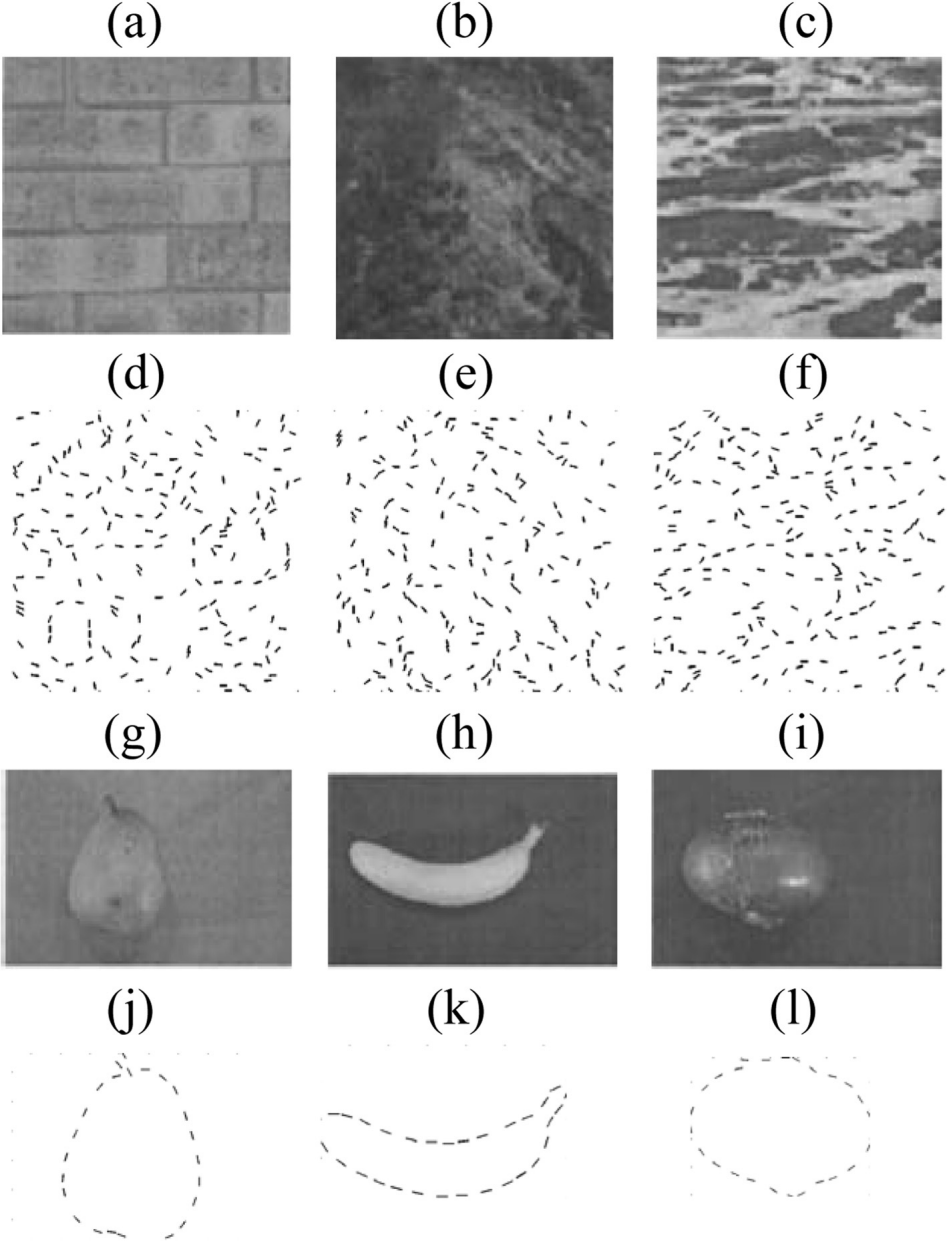
**Real images constructing objects and backgrounds. (a)** brick; **(b)** terrain; **(c)** water; **(d)**-**(f)** edge images of (a)-(c); **(g)** pear; **(h)** banana; **(i)** red onion; **(j)**-**(l)** edge images of (g)-(i).

Our purpose in the next experiment was to test the saliency detection performances on a collection of ”real images” under different SNRs(signal-to-noise ratios). We constructed testing images from pairs of real images. Three fruits^b^ were selected as the objects (Figure 7(g)-(i)), and six natural texture images from the MIT Media Lab texture database were selected as the background (three of which are given in Figure 7(a)-(c)). The Canny edge detector was applied to each object and texture background to yield edge images (Figure 7(d)-(f), (j)-(l)).

**Figure 8:**
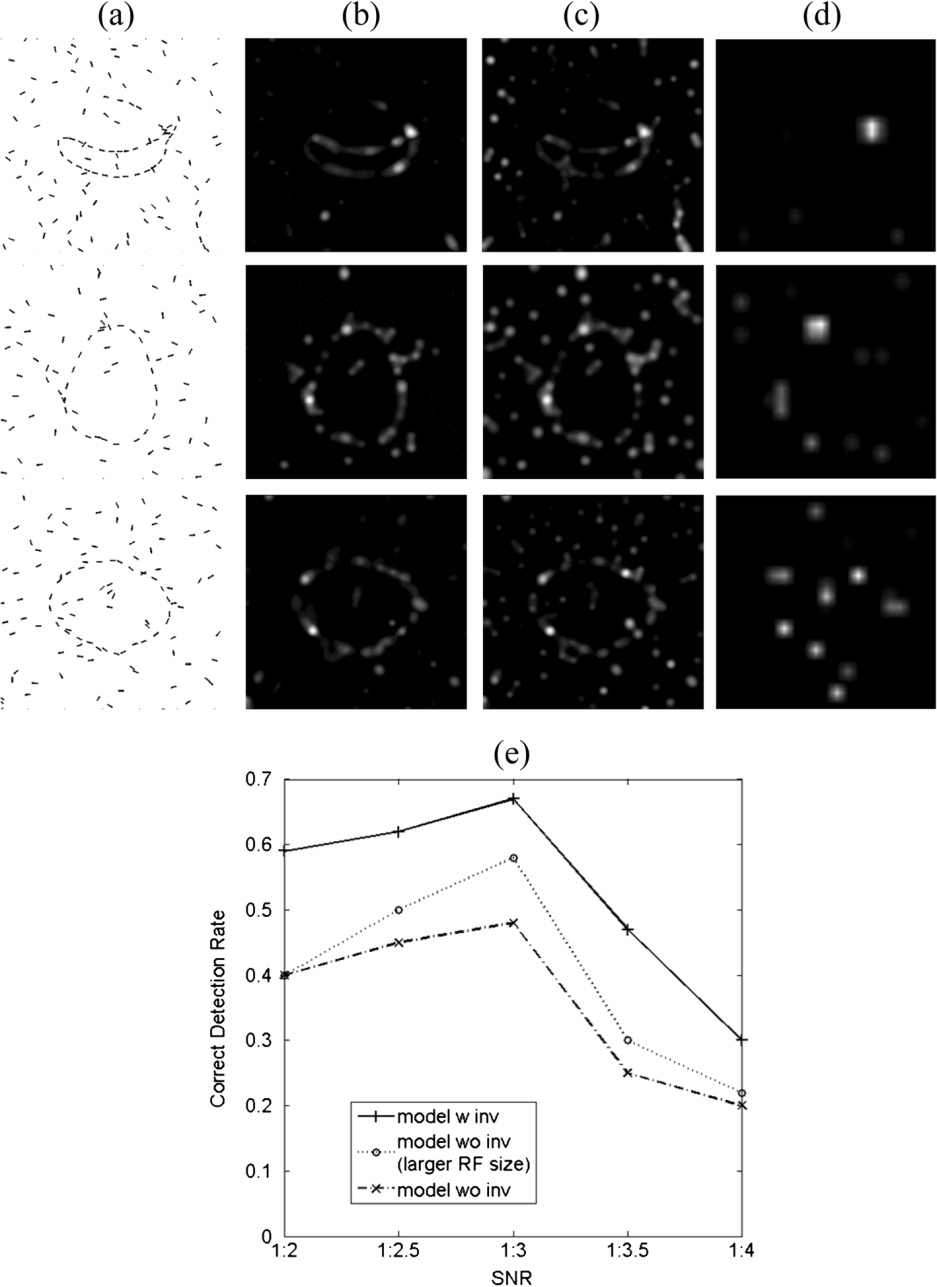
**Saliency detection performances on real images under different SNRs (a) 3 examples of testing images. ***Up*: the banana in terrain background at the SNR 1:3, *mid*: the pear in brick background at the SNR 1:2.5, *down*:the red onion in water background at SNR of 1:3.5; **(b)** saliency maps of the attention model with invariant coding; **(c)** without invariant coding; **(d)** saliency maps of the Saliency Tool. In a saliency map, the more salient part is indicated by the brighter and whiter region; **(e)** plot of the correct detection rates versus SNRs. Larger RF size improves the performance of the model with invariant coding, which coincides with findings in neuroscience.

A testing image was constructed by inserting an object edge image into the center 32×32 region in a 64×64 edge image of a texture (Figure 8(a)). An object consists of approximately 30 segments, and a texture edge image was undersampled at different scales to produce the background patterns consisting of different numbers of segments. We kept the number of segments in the objects fixed and changed the number of segments in the background to obtain testing images with different SNRs. Under each SNR, we estimated the correct detection rate on 18 images (a combination of an object from 3 fruits and a background from 6 texture patterns) to get statistical results of saliency detection on these real images.

Given a testing image in which the object consists of *m* edges, we defined a correct detection as the object edges which account for not less than 70% the first *m* most salient edges. It can be computed by a Bernoulli binomial probability distribution that the random probability is not higher than 0.016^c^. Several examples were shown in Figure 8. It was a challenging task for the segments along the object silhouettes were not uniformly spaced and the segments in backgrounds were correlated (possibly formed colinear or co-circular structures). The model with invariant coding outperformed the model without invariant coding at the same set of parameters. When the RF size was tuned to be larger in the model without invariant coding, it performed better. Saliency Tool failed in the task. A quantitative comparison of the correct detection rates versus SNRs was plotted in Figure 8(e).

From the plot, we can see that the correct detection rate first slightly increases and then dramatically decreases as the SNR decreases. This result can be explained in the following way. When the number of background segments is small, their saliency may surpass that of objects, for the background segments are so sparse that they contrast strongly with surroundings. When the number of background segments is large, their probabilities of forming collinear or continual structures increase, leading to many local maxima in saliency maps and thus disturbing the detection of objects. In our experiments, the correct detection rate reaches the maximum at the SNR of 1:3.

Why did the attention models differ in saliency detection? This could be caused by two factors. First, the sizes of receptive fields are enlarged after pooling, so some discrete segments or end points are easier to be perceived as a whole. It has been reported that larger receptive fields facilitate object search in complex scenes [23]. An improvement in the performance of the model without pooling (i.e. the model without invariant features) but with a larger RF size also supports this factor.

Second, invariant coding makes smoothly varying stimuli evoke consistent responses, thereby enhancing the contour and facilitating perception of a structure from a cluttered background. This phenomenon is known as the contour completion, which is achieved since neurons with similar preferred orientations enhance each other when they are collinear (smoothly varying in orientations), and suppress each other when they are nearly orthogonal [15]. This kind of interaction is also reported in [9] as neuronal responses that are modulated by the presence of stimuli outside of classical receptive fields.

### Effects of overcomplete representation on saliency detection

In this section, we used natural image datasets to test the effects of overcomplete representation on saliency detection. The experiments include two parts. In the first part, we learnt filters from the same set of images as used in the last section. The numbers of filters are set to be 100, 196, 392, and 576, respectively, and correspondingly 16, 25, 50, and 64 invariant feature descriptors by pooling the filters are selected. The testing dataset is collected by Bruce et al. [24], which includes 120 color images and eye movements from 20 observers when they view these images. The human eye tracking data can be used as a physiological basis to compare with the saliency maps obtained from attention models. In the second part, we selected 50 images from the Weizmann dataset [25] and collected eye tracking data of 15 viewers on these images. All these images show targets in cluttered texture backgrounds. 30 images out of the 50 images were used for training filters, and the 20 images left were used for testing.

Each image was scaled to 341×256 pixels. In learning by the PCICA, we chose, for example, 16 neighbourhoods (pools) of 5×5 from the 10×10 topographic array (composed of 100 filters) where the size of each filter is 16×16. Two neighbourhoods were overlapped by two filters both horizontally and vertically. Torus grid was used, that is, next to the nethermost filter is the corresponding uppermost filter, and next to the rightmost filter is the corresponding leftmost filter. In testing, we used the 16 sets/pools of filters (each set was composed of 5×5 filters) to extract features from the 341×256 images. Next, we pooled the 25 filtered results in each set into a separate feature map. After performing surround suppression within each feature map, 16 conspicuous maps with a size of 341×256 were obtained. Finally, a single 341×256 saliency map was formed by combining 16 conspicuous maps.

**Figure 9:**
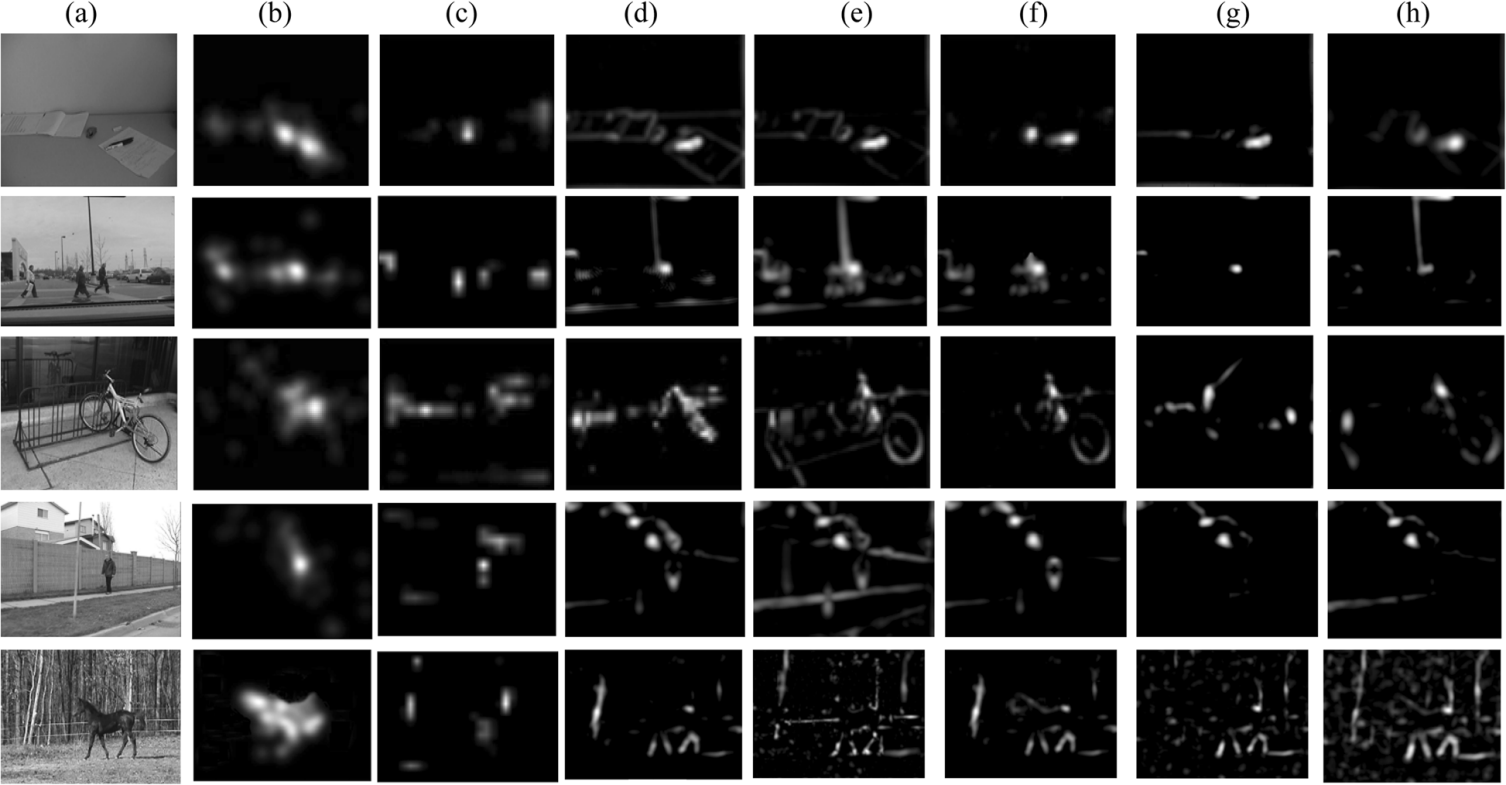
**Saliency maps of attention models with different numbers of bases. (a)** source images; **(b)** human eye tracking; **(c)** saliency maps of Itti’s attention model; (d) saliency maps obtained by the attention model with 100 bases (16 feature maps); **(e)** with 392 bases (50 feature maps); **(f)** with 576 bases (64 feature maps); **(g)** saliency maps of a fully connected network (392 bases, 1 feature map); **(h)** saliency maps of a randomly connected network (392 bases, 50 feature maps). The attention models in this paper simulate bottom-up saliency detection. Thus, their results are not always identical to the results of human eye tracking, which sometimes involves top-down attention. In the last row, our model with 576 filters (64 invariant features) acts like a contour extractor that can suppress textures. It detects most contours of the target despite the strongly cluttered background. All the filters are learned from the same training dataset.

The model of Itti and Koch (Saliency Tool) uses Gaussian pyramids with 9 scales and 4 orientations. At each orientation angle, this model pools primitive features obtained from Gabor filters of different phases. In this experiment, we set the number of phases at 4 for computational efficiency and also because this value does not substantially influence the results. To make the results of the two models comparable, we only selected the intensity and orientation channel for the Saliency Tool to form saliency maps. Altogether, Saliency Tool uses 144 filters and combines 30 feature maps (4 orientation features, 1 intensity feature, and 6 centre-surround difference maps for each type of feature; see [26] for details). This setting is close to that of our model with 196 filters^d^ and 25 feature maps to be combined. Several examples are given in Figure 9. 

We computed the receiver operator curve area (ROC area), a common measure in signal detection [27], to compare the performances of different models with observations from humans, and list the ROC scores in Table 1. The larger score means better consistency with human observers. Considering that the filters and invariant feature descriptors are learned from gray images and no color information is encoded, the attention models based on these can detect saliency mainly caused by intensities and orientations. Therefore, in the Bruce dataset, we transformed the color images into gray images and removed the images whose saliency was only caused by color contrasts. The remaining 82 images were used for testing. In the Weizmann dataset, all images are gray images. 

**Table 1:** ROC scores in different datasets

		**Basic number**	**ROC**			**Basic number**	**ROC**
		**(feature maps)**				**(feature maps)**	
		100(16)	0.5722			196(25)	0.7104
	our	196(25)	0.6427		our		
Bruce	model	392(50)	0.6830	Weizmann	model	256(36)	0.7282
data		576(64)	0.6864	data			
	Sal Tool	144(30)	0.6717		Sal Tool	144(30)	0.6233
	ful net	392(1)	0.5913		ful net	256(1)	0.4947
	rand net	392(50)	0.5965		rand net	256(36)	0.5011

As shown in Table 1, the saliency detection accuracy improves as the number of the basis set increases. This may be explained by the fact that the more overcomplete basis set describes features of images (namely frequencies, orientations, and positions) more adequately [28]. It covers almost the whole frequency (orientation, phase) space for natural images and encodes primitive features as well as shape features.

In the Bruce dataset, when the number of a basis set is too small, such as 100 bases pooled into 16 invariant feature descriptors, they cannot describe an image adequately, resulting in great divergence from human detection. When the number of a basis set reaches 392 or 576, the change of ROC scores is tiny. This indicates that a 392 basis set (two times overcomplete basis set) is near saturation. The ROC score at this point is above that of Saliency Tool, which indicates the superiority of overcomplete basis set. The performances of the randomly connected network and the fully connected network are also listed. The performances of these networks are worse than our model, though the differences are not as significant as those in synthetic images.

The possible reasons for the difference between the results of synthetic and natural images can be analyzed from two aspects. First, synthetic images(which are specially designed) and natural images have different structures. In Bruce dataset, a considerable number of images have no uniformly distributed distractors and almost no collinear or cocircular segments. Saliency detection results for such images do not differ significantly in different networks.

Second, our model with invariant features yields a saliency map coding intensity and orientation, whereas the fully and randomly connected networks produce saliency maps only coding intensity. For the synthetic images used in our experiments, the intensity cannot differentiate targets from distractors∖noises (as their intensities are equal). Without orientation information, the targets cannot pop out. For most natural images from the Bruce dataset, the intensity contrast alone contributes greatly to the final saliency maps. Therefore, saliency detection results on the Bruce dataset do not differ substantially between our model and the fully∖ randomly connected networks.

However, our model with invariant features does show distinctions in certain kinds of natural images: the images where orientation plays a major role in saliency detection and the images where targets are in cluttered texture.

**Figure 10:**
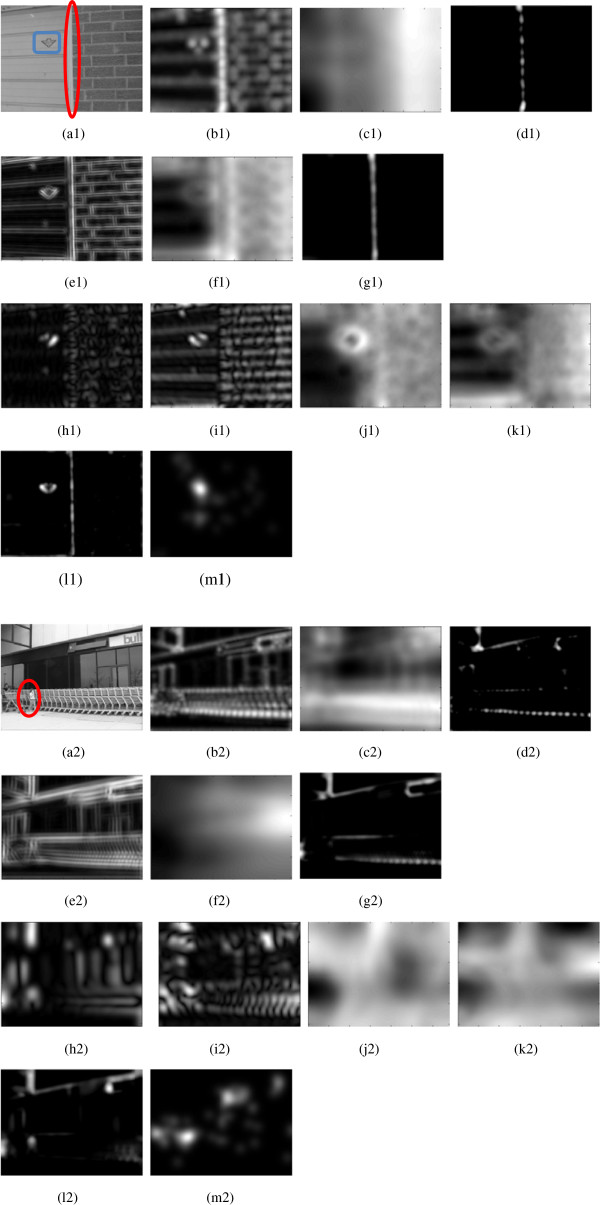
**Two examples where our model shows distinctions. (a1)** source image; **(b1)** the pooling result of the fully connected network, which does not discriminate different orientations, but shows intensity differences. After the surround inhibition shown in **(c1)**, only the part with the strongest intensity contrast is left in the saliency map **(d1)**. **(e1)** The pooling result of one of the random groups in the randomly connected network, which is similar to the case in the fully connected network; **(f1)** the surround inhibition performed on **(e1)**; **(g1)** the final saliency map. **(h1)** and **(i1)** two examples of pooling results in our model, which are specific to certain orientation angles; **(j1)** and **(k1)** corresponding surround inhibition; **(l1)** the final saliency map. **(m1)** human eye tracking. **(a2)** source image; **(b2)** the pooling result of the fully connected network, where the saliency of the pedestrian is too weak to be discriminated from the cluttered background. After the surround inhibition shown in **(c2)**, only the part with the strongest intensity contrast is left in the final saliency map **(d2)**. **(e2)** the pooling result of one of the random groups in the randomly connected network, which is similar to the case in the fully connected network; **(f2)** the surround inhibition performed on **(e2)**; **(g2)** the final saliency map. **(h2)** and **(i2)** two examples of pooling results in our model, which are specific to certain orientation angles, and the saliency of the pedestrian is strong enough to support the pop-out; **(j2)** and **(k2)** corresponding surround inhibition; **(l2)** the final combined saliency map; **(m2)** human eye tracking.

An example showing orientation plays a major role in saliency detection is given in Figure 10(a1)-(m1). In the fully connected and randomly connected network, only the vertical line (marked by a red box) is detected as salient. In our model, the triangle sign (marked by a blue box) is also detected as salient, which is more consistent with human perception. The vertical line is salient because of the strong intensity contrast between its left and right regions, whereas the orientation contrast contributes more than intensity to the saliency of the triangle sign.

An example showing the pop-out of targets in cluttered texture is given in Figure 10(a2)-(m2). In the fully connected and randomly connected network, a pedestrian (marked by a red box) in a cluttered background cannot pop out. In our model, the pedestrian is detected as salient, which is more consistent with human perception.

**Figure 11:**
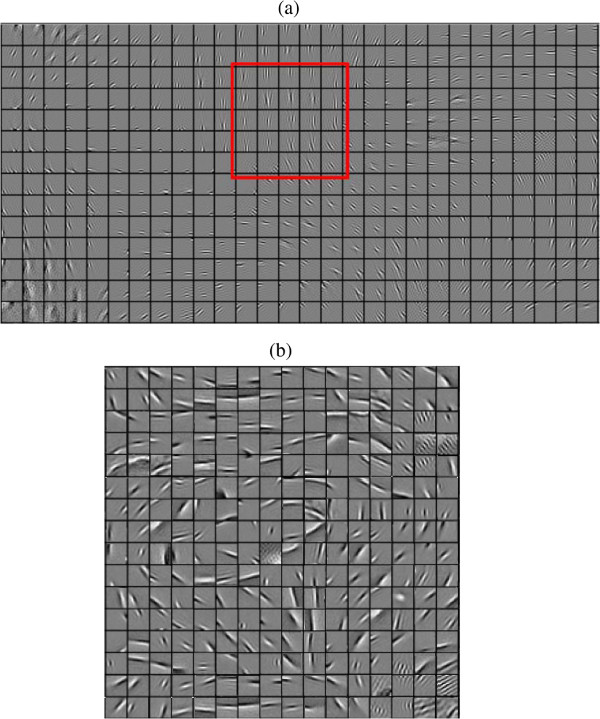
**Overcomplete bases obtained by PCICA.** A clear topography emerges from the two maps. Though both sets consist of Gabor-like filters, filter patterns and distributions learned from different datasets appear to be different, particularly in their average length and frequency. **(a)** the basis set that is used in most experiments (except the experiment on Weizmann dataset). It is learned from natural images. An example showing the filters in neighborhood slowly change their properties as marked within a red square; **(b)** the basis set that is learned from Weizmann dataset. Some bases are similar to receptive fields of curvature-selective, end-inhibition, side-inhibition and texture-selective cells.

As to the Bruce dataset that is used in our experiments, the images where our model with invariant features shows distinctions are few. Thus the ROC scores have no substantial differences among different networks.

we selected 50 images from the Weizemman dataset. All the selected images show targets in backgrounds with cluttered texture. 30 images were used for training filters, and the 20 images left were used for testing. The filters learned from this training set was given in Figure 11(b). The performance of different networks on this dataset were listed in Table 1. We can observe divergences between our model and other networks. This experiment shows that our model with invariant coding can fully exhibit its superiority, given special datasets and filters learnt from task-dependent images.

### Relations between our model and Itti’s Saliency Tool

Our model uses orientation and intensity feature to produce saliency maps and calculates within feature competition and combination in a similar manner to that of Saliency Tool. From this point of view, our model may be mistaken as a special case of Itti and Koch’s model (when constrained to use only orientation information). However, the two models are distinct from one another, as mainly manifested in the following aspects:

First, the model by Itti and Koch constructs Gabor filters in 4 orientations and 9 scales to extract primitive features, whereas ours learns an overcomplete set of filters from training images. Thus, Saliency Tool works in an unsupervised way, while our model can be extended to a supervised one. Though the learning process is more complicated and time consuming than direct construction, it greatly improves performance for it provides a specialised model (the filters are learned by providing the system with examples of targets to be detected). Such works [29,30] have been reported to improve saliency detection by learning feature descriptors and by training classifiers with positive and negative samples.

**Figure 12:**
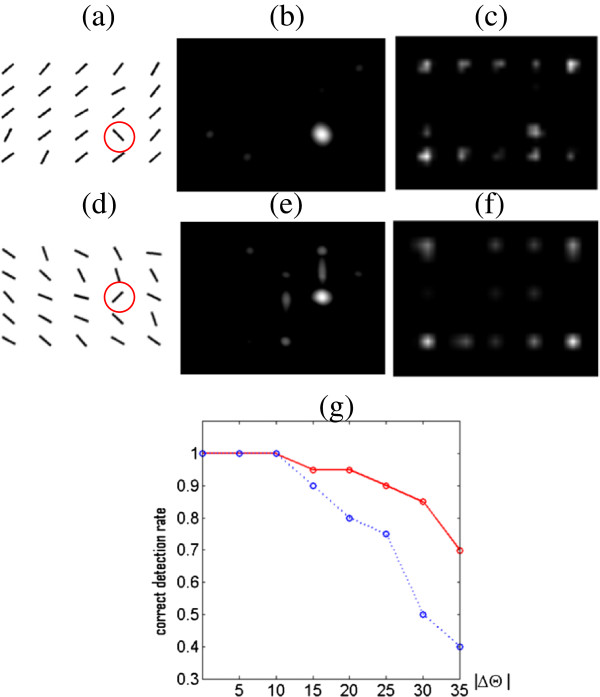
**Invariance to orientation. (a)** an example of images where orientation noises are in [−20°, 20°] with uniform probability; **(b)** saliency map of our model for (a); **(c)** saliency map of Saliency Tool for (a); **(d)** an example of images where orientation noises are in [−30°, 30°] with uniform probability; **(e)** saliency map of our model for (d); **(f)** saliency map of Saliency Tool for (d); **(g)** statistics of correct detection rate vs. range of orientation noises |*Δθ*| in distractors (that is, when the orientation of the target is *θ*_*t*_, the orientations of distractors vary in [*θ*_*t*_ + *pi*/2−*Δθ*,*θ*_*t*_ + *pi*/2 + *Δθ*]). For each |*Δθ*|, we test 20 randomly generated images. The target is marked by a red circle. The results of Saliency Tool are got by only using orientation information. Note that since orientation difference between the target and distractors is *pi*/2, |*Δθ*| should not exceed 45°.

Second, the orientation features obtained by the two models are different. At each orientation angle (0°, 45°, 90°, 135°), Saliency Tool pools Gabor filtering results at different phases by a sum of the absolutes (∑||). The default is pooling a pair of Gabor responses with orthogonal phases. In our model, filters in the same pool have similar orientations (smoothly changed) and different phases. Hence, our model obtains orientation invariance aside from location invariance. When the orientation of input data slightly changes, the features do not change greatly. By contrast, Saliency Tool is location invariant but not rotation invariant. A small change in input orientation changes the representation significantly. To test this point, we reused the visual search task mentioned in Figure 4 and listed the performances of the two models in Figure 12. When all distractors were in the same orientation, the two models performed equally well. As the orientation noises in the distractors increased (that is, the distractors increasingly varied in orientations), the correct detection rate of Saliency Tool greatly dropped, whereas the correct detection rate of our model did not change significantly. The two models differ in terms of the robustness to minor orientation variations in input. In Saliency Tool, the orientation contrast of the target to distractors is likely to be weakened or be entirely lost (the worst situation) when variations among distractors increase. It is so sensitive to this variation that the correct detection rate drops.

However, a feature descriptor designed to be insensitive to great variations in input may be unfavourable especially for object recognition.

## Conclusion

To determine which factors in coding models affect saliency detection, we construct a coding model satisfying neurobiological constraints to provide input to the bottom-up attention model. The model is plausible in neurobiology because it provides an overcomplete representation for stimuli, obtains invariant features by a hierarchical structure, and models feature competition through nonclassic receptive field inhibition. We analyse different network structures and parameters as well as the underlying factors that influence performance.

More specifically, invariant coding improves robustness to noises and distractors, and improves the ability of detecting salient structures, such as colinear and co-circular structures that satisfy some perceptual organization rules. This phenomenon is also found in physiological experiments [16]. We have also shown that overcomplete basis set encodes a rich repertoire of natural image features, so it can improve saliency detection accuracy.

Given the state-of-the-art models by Itti and Koch, and Zhaoping Li, our work provides a learning scheme that can be easily extended to a supervised model. By learning from examples of targets to be detected in a specific task, the performance is expected to be improved. The detailed behavioural analysis for parameters is of reference to a construction of a good system.

In summary, our results suggest that hierarchical invariant coding and overcomplete representation are general principles in visual attention and possibly in other perceptual systems. In the future, we will extend our attention model to work in a supervised method, involve multiscale techniques for adaptation to image resolutions, and to include other features (like colour) into saliency detection.

## Methods

Before applying our model to synthetic or natural images to yield saliency maps, we obtained a basis set by training whitened image patches according to the PCICA algorithm. A brief summary of the training process was given in “Results and discussion” section (refer to [22] for details). A set of 392 overcomplete bases of 16×16 learned from natural images was listed in Figure 11(a). In the preprocessing, all the 16×16 training patches were whitened. That is

(1) I←WI

where *I* is a matrix consisting of all patches. *W* is the whitening matrix computed by

(2) $$W=VU^{-\frac{1}{2}}V^{T}$$

where *U* and *V* are the eigenvalue matrix and eigenvectors from *II*^*T*^=*VUV*^*T*^.

The dimension of all patches was decreased from 256 to 196 by Principle Component Analysis. As the number of bases is twice the dimension of a basis, it is designated as two times overcomplete bases. The bases learned from natural images show clear topography. Orientations, frequencies, and locations of all the filters smoothly vary, forming a globally and topographically ordered array. Properties of filters in neighborhoods are similar.

The workflow of the testing process was described in Figure 1. Next, we break down the testing process into detailed subsections.

### First layer - primitive features extraction

Given a set of overcomplete topological basis vectors {*Φ*_*i*_} learned from natural images by the PCICA algorithm, we compute their responses to an image *I*(*x*,*y*) by convolution

(3) $$SF_{i}(x,y)=\frac{\Phi_{i}^{T}}{∥\Phi_{i}∥}*I(x,y)$$

As each basis responds optimally to a specific frequency, phase, and orientation, local primitive features similar to simple cell responses are encoded by formula (3). Then *SF*_*i *_is rectified by the absolute and sigmoid function to limit its range between 0 and 1.

(4) $$SF_{i}\leftarrow \text{sigmoid}(|SF_{i}|)$$

A similar rectification is implemented by the hyperbolic tangent and absolute function in [12].

### Second layer - invariant features representation

Invariant features are obtained by organizing the responses of topological bases in the same neighbourhood with the pooling operations. When extracting invariant features from an input images, the neighborhood size should be the same as that in learning topographic bases by the PCICA. An example is denoted by a red box in Figure 11. For the two times overcomplete bases mentioned above, we set the size of neighborhood to be 5×5 and two adjacent neighbourhoods overlap by two bases in both rows and columns. In this way, invariant feature descriptors *Ω*_*j *_are obtained and each of them consists of 25 bases. Bases in the same descriptor correlate strongly, while bases in different descriptors usually correlate weakly.

The pooling cannot avoid some bases with strong differences being grouped into the same neighborhood, so a further refinement is needed. In every descriptor, each basis *ϕ*_*i *_is compared with the basis located at the center *ϕ*_*c *_by a similarity measure $\rho_{\phi_{i}\phi_{c}}$, which is defined as a high-order correlation between the features encoded by the two bases

(5) $$\rho_{\phi_{i}\phi_{c}}:=\text{corr}(SF_{i}^{2},SF_{c}^{2})$$

**Figure 13:**
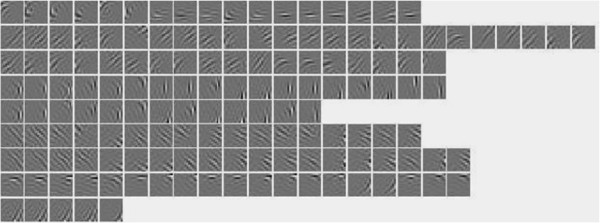
**Some examples of invariant feature descriptors obtained by pooling and then refining.** Each row lists a descriptor *Ω*_*j *_in which orientations and frequencies are similar but phases are different.

We compute the high-order correlation as the method given in TICA [21]

(6) $$\text{corr}(SF_{i}^{2},SF_{c}^{2})=\frac{E(SF_{i}^{2}SF_{c}^{2})-E(SF_{i}^{2})E(SF_{c}^{2})}{\sqrt{D(SF_{i}^{2})}\sqrt{D(SF_{c}^{2})}}$$

where *E*() and *D*() denote the expection and variance function, respectively. *SF*_*i *_was computed by using the formula (3). In a descriptor, the bases correlating weakly to the center basis, indicated by $\rho_{\phi_{i}\phi_{c}}$ under some threshold, are removed. After this processing, the filters in the same descriptor have similar properties. Figure 13 shows nine examples of invariant feature descriptors obtained by pooling and then refining with a correlation threshold of 0.1. 

After the invariant feature descriptors are determined, the responses *SF*_*i*_(*xy*) of all filters *ϕ*_*i *_belonging to the same descriptor *Ω*_*j *_are pooled

(7) $$CF_{j}(x,y)=H\left(\underset{\phi_{i}}{\sum }SF_{i}^{\beta }(x,y)\right)\;,\;\;\phi_{i}\in \Omega_{j}$$

where *CF*_*j*_(*xy*) is the output of an invariant feature descriptor. *H*() and and *β*are related to the pooling nonlinearity. A number of choices might be used to quantify this nonlinearity. A popular one is *H*() as a square root function and *β*=2. It was reported that such a choice was optimal for TICA and ISA [20,21].

As the orientations and frequencies of filters belonging to the same descriptor vary smoothly, we can obtain invariance by this pooling operation. It is biologically plausible that a bank of receptive fields at nearby locations on one level are organized to provide input to a receptive field on a higher level [31,32]. By pooling, the size of a receptive field on a high level is enlarged compared with the one on a low level, and its robustness to changes is increased as well. Simple cells and complex cells in the V1 area are an example. Receptive fields of simple cells overlap with each other, and those with similar properties pool to form receptive fields of complex cells.

Although topology is not indispensable for calculating invariance, it is used in numerous models (TICA, temporal product network, IPSD, deep networks) to provide an organised group for an upper layer to obtain invariance. A popular alternative solution is to keep the unarranged layout in a low layer and select the pooling group by some similar metric defined on filter responses [33]. We investigate topographic filters in this paper because of their biological plausibility (complex cells in V1 do take on a topographic layout) and because the PCICA algorithm is computationally efficient and insensitive to tuning parameters.

### Within feature competition

After an image is encoded by invariant feature descriptors, the part that differs the most from its surroundings in a feature map is selected as a candidate for salient objects. The competition in this process is within a feature map. To discriminate this competition from the inter map competition afterwards, we refer to this competition result as conspicuous maps. Motivated by neurobiology, we obtain a conspicuous map by modelling suppression between neurons with a DoG operator. This kind of suppression is also called the nonclassic receptive field suppression which was observed in the primary visual cortex of the macaque monkey [34-36].

(8) $$\text{DoG}(x,y)=\frac{1}{2\Pi \sigma_{\mathrm{ex}}^{2}}e^{-\frac{x^{2}+y^{2}}{2\sigma_{\mathrm{ex}}^{2}}}-\frac{1}{2\Pi \sigma_{\text{inh}}^{2}}e^{-\frac{x^{2}+y^{2}}{2\sigma_{\text{inh}}^{2}}}$$

where *σ*_*ex *_and *σ*_*inh *_indicate excitation and inhibition bandwidth, respectively. In experiments, the value of *σ*_*ex *_denotes the times of excitation bandwidth with respect to the size of filters. For example, if *σ*_*ex*_=2, excitation bandwidth is actually set to twice the size of filters. For the model with invariant features, this kind of suppression acts on the output of invariant feature descriptors(pooling results), forming a conspicuous map

(9) $$s_{j}(x,y)=|CF_{j}(x,y)-\alpha (CF_{j}⊗\omega )(x,y){|}_{\geq 0}$$

(10) $$\omega (x,y)=\frac{1}{∥\text{DoG}{∥}_{L1}}|\text{DoG}(x,y){|}_{\geq 0}$$

where *α* is a coefficient that adjusts the strength of suppression, ⊗ denotes convolution, ||_≥0 _remains unchanged if inputting is positive, and outputs zero if inputting a negative or a zero. ∥*DoG*∥_*L*1_ is the *L*1 norm.

For the model without invariant features, this kind of suppression directly acts on the rectified output of convolutions between filters and an input image.

### Combination strategy

Finally, conspicuous maps are integrated into a saliency map by a certain combination strategy. The simplest combination strategy is summing up all feature maps after normalization (for example, normalize the data into the same range). However, the strategy will encounter a severe problem. The maximum in one of the feature maps is very likely to be weakened by the maxima in other maps or noises, or even completely lost. Several improved strategies such as weighted sum are relatively more robust to noises.

**Figure 14:**
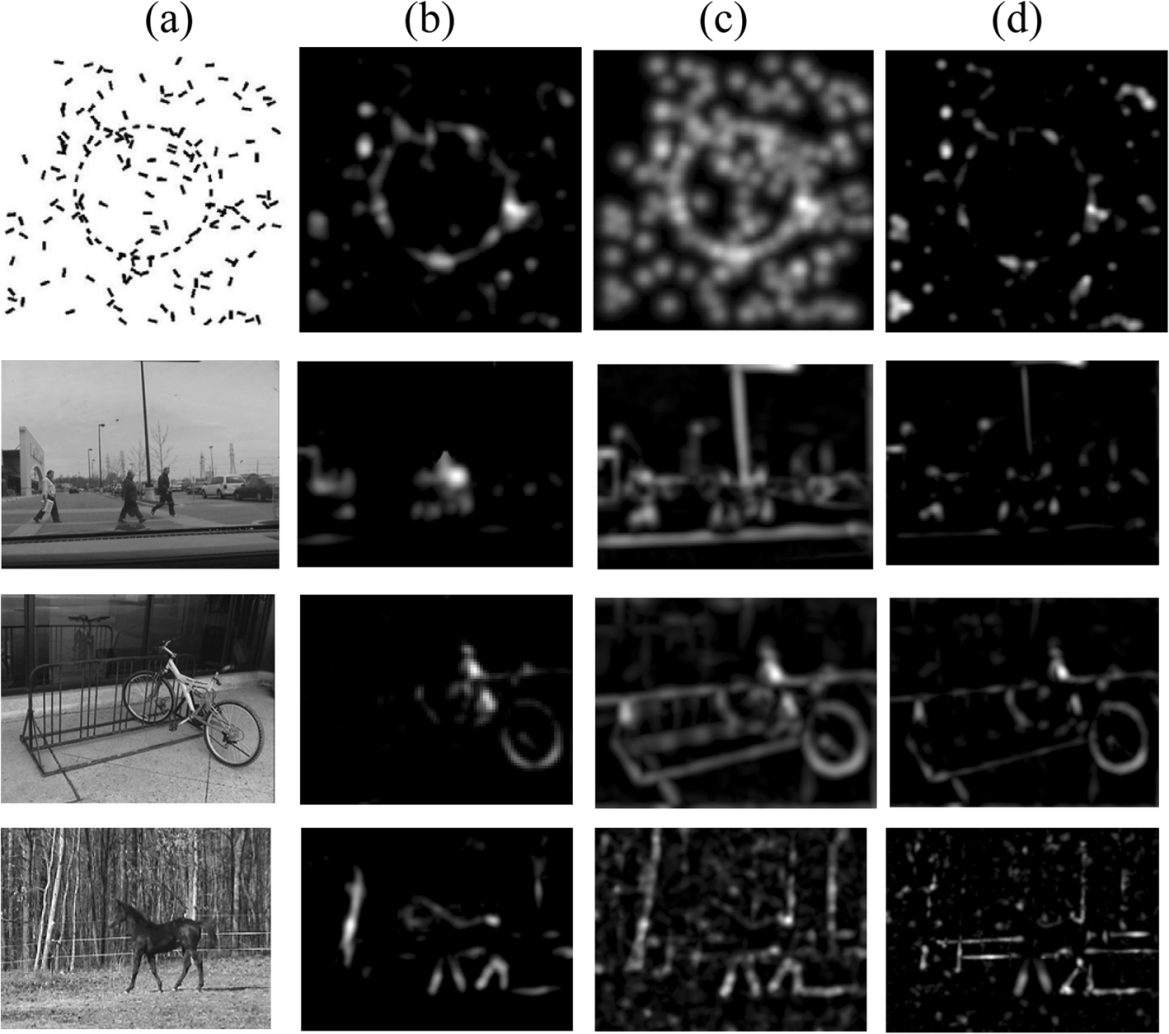
**Comparison of different combination strategy. (a)** source images; **(b)** result of our combination; **(c)** result of combination by sum; **(d)** result of combination by max.

Zhaoping Li [7] proposed that a single max operator (over all neurons regardless of their preferred input features) is more plausible than the sum in feature integration. Psychophysical tests of their V1 saliency hypothesis on composite patterns are given in their report as an example to explain how the max operator works when sum fails. While the max operator works well in some psychophysical patterns, it is not highly robust to distractors that are frequent in natural images. The max operation is independently performed at each map location. The saliency value of a distractor is probably a local maximal in a feature map. If this local maximal is also the maximal among all feature maps at this location, it will appear in the saliency map. Thus, the max combination sometimes yields a saliency map in which many locations are peaks, as shown in Figure 14. 

In this paper, we adopted a combination strategy of iteration [37]. At each iteration, a given feature map *s* is subjected to the following transformation

(11) $$s\leftarrow |s+s⊗\text{DoG}-C_{\text{inh}}{|}_{\geq 0}$$

where *DoG* is a two-dimensional difference of the Gaussian operator. *C*_*inh*_ is a constant inhibitory term and puts a small bias to slowly suppressing areas where excitation and inhibition balance almost exactly [37]. After a few iterations, the initial maximum in a feature map will be further strengthened, and the initial non-maxima will be further suppressed. As a result, the differences between the maximum and the non-maxima are amplified.

When the number of maps to be combined is less than 10, we iterate them as aforementioned and sum up the results to obtain a saliency map. When the number of maps is large, for example, 64 maps by pooling 576 overcomplete bases, a direct application of the iteration strategy faces a severe signal-to-noise problem. Thus, we provide a modified version.

We first compute a similarity matrix (defined in formula (6)) for all conspicuous maps. Then, we use the K-means algorithm (or any other spectral clustering method) to organise the maps into N clusters according to the similarity between pairs of them. Within each cluster, we pool all maps into a single map (average pooling is used in the experiments) and thus obtain N separate maps, one for each cluster. Next, each N map is subjected to the iteration described in formula (11). Finally, we obtain the saliency map by summing up the N iterated results.

Computing the similarity matrix for conspicuous maps may be time consuming, especially when a conspicuous map is in high resolution. We solve this problem by subsampling. We typically use *N*=8, though our results are not too sensitive to this parameter.

Several examples using the aforementioned combination strategy are given in Figure 14. For comparison, the results of the max and the simple sum are provided as well. Iteration produces much sparser maps where most of the noisy activities are strongly suppressed. A simple sum yields poor performance. Although the iteration performs best, it has the largest number of free parameters and complex processes. The parameters depend on object size, type of image, or on top-down influences.

## Appendix

### Appendix 1 Influences of different parameters in our model

**Figure 15:**
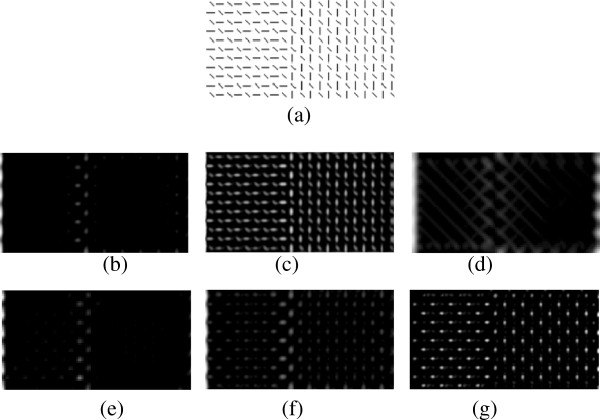
**Influences of different parameters in our model with invariant features on a composite stimulus. (a)** source image; **(b)** result of optimized parameters; **(c)** smaller RF size in layer 1, which fails in detecting the target; **(d)** bigger RF size in layer 1, which produces a strange result; **(e)** smaller RF size in layer 2, which does not influence the result significantly; **(f)** smaller coefficient *α*which produces less strong suppression, but still detects the target as the most salient bars; **(g)** smaller excitation bandwidth *σ*_*ex*_, which fails in detecting the target.

**Figure 16:**
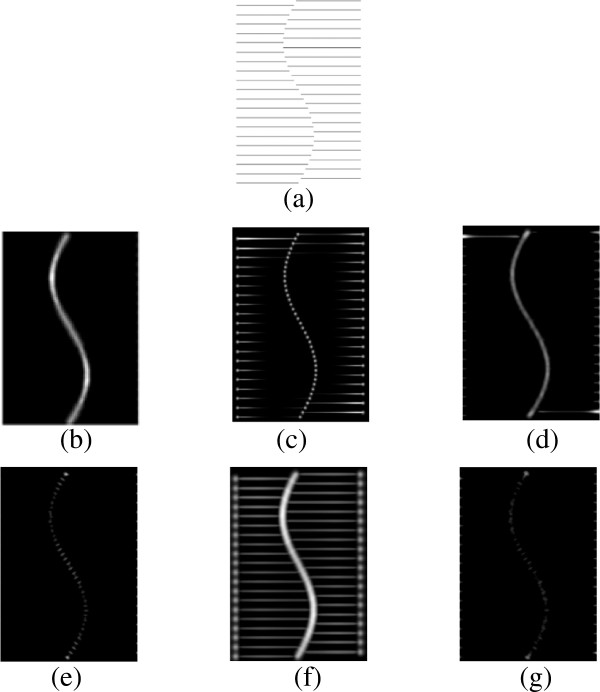
**Influences of different parameters in our model with invariant features on an illusory contour. (a)** source image; **(b)** result of optimized parameters; **(c)** smaller RF size in layer 1, which still detects the s contour but in a less continuous way (discrete points can be seen on the s contour); **(d)** bigger RF size in layer 1, which produces a more continuous s curve and no obvious border effects; **(e)** smaller RF size in layer 2, which does not influence the result significantly (discrete points can be seen on the s contour); **(f)** smaller coefficient *α*which produces less strong suppression; **(g)** smaller excitation bandwidth *σ*_*ex*_, which detects a weak s contour.

The influences of parameters on saliency detection are relevant to the type of stimuli and to the difficulty of the task. We can see that the influences on a composite stimulus (Figure 15) and on an illusory contour (Figure 16) are different. Generally speaking, for our model with invariant features, the first layer RF size is highly relevant to the resolution of the image and to the texture density. The second layer RF size does not substantially affect the performance. The coefficient *α*that adjusts the suppression strength cannot be too small (responses to distractors cannot be completely suppressed) and too large (responses to targets are also suppressed). The excitation bandwidth *σ*_*ex *_in the DoG that is used in within the feature competition leads to a degraded performance when it is smaller than the layer 1 RF size.

**Figure 17:**
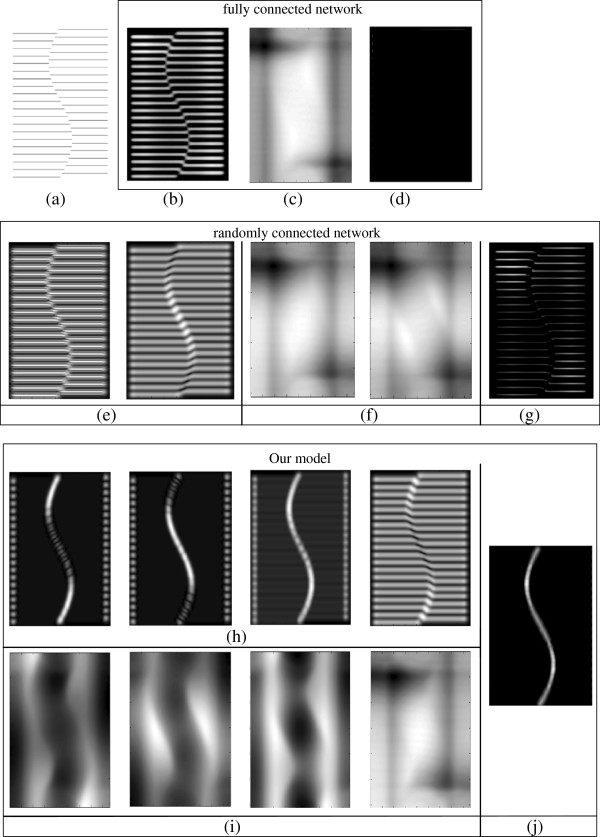
**Comparison of different networks on illusory contour. (a)** source image; **(b)** pooling result of fully connected network, which is almost a copy of input image since all features are reserved; **(c)** inhibition term of fully connected network which is almost uniform in the whole space; **(d)** saliency map of fully connected network; **(e)** two examples of pooling results of randomly connected network; **(f)** inhibition terms of randomly connected network corresponding to (e); **(g)** saliency map of randomly connected network; **(h)** four examples of pooling results of our model which describes features in specific orientations ; **(i)** inhibition terms of our model corresponding to (h); **(j)** saliency map of our model.

We test four layer 1 RF sizes, namely 8×8, 12×12, 16×16 and 20×20. Note that the learning time of the PCICA algorithm increases with the increase of this size. When the size is bigger than 20×20, our machine will run out of memory. As to the stimuli in Figure 17, 8×8 is too small to detect the targets. The size of 20×20 produces a very strange result for the composite stimulus, while it produces a better result for the illusory contour. When the stimulus is a uniformly distributed texture like the two examples, the layer 1 RF size should not be smaller than the average intervals between adjacent elements.

We compare three layer 2 RF sizes, namely 3×3, 5×5 and 7×7. They do not affect the performance substantially. Adjusting layer 2 RF size can be done in two ways.

One is to determine the size during the learning of the topographic filters by the PCICA. Larger sizes make the filters in each pool increasingly similar. If the size is reduced to 1×1, then it is equivalent to sparse coding. In this way, a larger size is beneficial to invariant representation.

The other is to determine the size when we extract features using the learnt filters. Given a fixed set of learnt topographic filters, we set (by default) the size of the subregions to obtain invariant features to be the same as the neighbourhood size in learning topographic filters by the PCICA. The size of the subregions can also be adjusted around that scale if necessary. In this way, the size of the subregions to obtain invariant features is the actual second layer RF size. Then, the subregions are subjected to refinement (computing the nonlinear correlations and setting a threshold to remove outliers according to equation (6)). If the second layer RF size is larger, the possibility of the RFs belonging to the same subregion but with strong differences becomes larger. Therefore, after removing the RFs with strong differences, the quantity of the remaining RFs is still close to that of the smaller second layer RF size. For example, when the second layer RF size is 3×3, the average number of RFs in a subregion/pooling group after refinement is 8. When the second layer RF size is 5×5, the average number of RFs in a subregion after refinement is 11. In this way, the second layer RF size does not influence the final results substantially.

For the coefficient *α*, we use three values: *α*∈{1.2,2.2,3.2}. Generally, a coefficient that is too small cannot completely suppress the responses to distractors, and a coefficient that is too large probably suppresses all responses including those to the target. Both of these situations lead to relatively bad performance.

For the excitation bandwidth *σ*_*ex *_and inhibition bandwidth *σ*_*inh*_, we use three values *σ*_*ex*_∈{0.35,1,2}, and keep the ratio $\frac{\sigma_{\text{inh}}}{\sigma_{\mathrm{ex}}}=4$ just for simplicity. Note that the value of *σ*_*ex *_denotes the times of the excitation bandwidth with respect to layer 1 RF size. No substantial change is observed for the bigger *σ*_*ex*_, but a degraded performance is observed at *σ*_*ex*_=0.35, i.e. a excitation bandwidth smaller than the layer 1 RF size.

### Appendix 2 Comparison of different networks on illusory contour image

A fully connected network produces (almost) blank results. The pooling result is shown in Figure 17(b), which preserves all information from the source image in a blurred version. This result can be expected because the filters in the first layer cover all orientations, phases, and frequencies. The pooling output of all these filters produces almost an exact copy of the original one. Then, the inhibition term (convolution of the pooling result with DoG) is shown in Figure 17(c), which is almost equally strong in all locations of the image plane. The final output (Figure 17(d)) is blank (zero) because all the information is inhibited.

The performance of a randomly connected network is similar to that of a fully connected network, for most of the random groups perform like a miniature fully connected network. This is also revealed from the network structures (Figure 2 and 3). If the size of the random group is equal to the size of the first layer, a randomly connected network is equal to a fully connected network. However, the probability does exist that most filters in the same random group may have similar orientations. Then, such groups can acquire invariant features, which may account for the fact that a randomly connected network sometimes performs better than a fully connected network.

For comparison, we list several pooled results of convolution in the same pool (Figure 17(h)) for our model with invariant features as well as the inhibition term within each pool (Figure 17(i)). The result by pooling convolutions in the same pool describes features in a specific orientation because filters in the same pool have similar orientations. The inhibition performed in such pools is completely different from that in fully connected networks (Figure 17(c)) because of the properties of the DoG.

## Endnotes

^a^http://research.ics.aalto.fi/ica/data/images/^b^http://www.cs.unm.edu/∼williams/saliency.html^c^Random probability computes $C_{10}^{7}{\left(\frac{m}{m+n}\right)}^{7}{\left(\frac{n}{m+n}\right)}^{3}$ where m is the number of object segments and n is the number of background segments^d^considering some filters are removed during refinement, the actual number of filters used is less than 196

## Authors’ contributions

ZQ designed the study and drafted the manuscript. ZS took part in designing the study and revised the manuscript. Wang Zhe took part in designing the experiments and analyzing the data. HY took part in revising the manuscript. All authors read and approved the final manuscript.
